# Misoprostol vs dinoprostone vaginal insert in labour induction: comparison of obstetrical outcome

**DOI:** 10.1038/s41598-021-88723-5

**Published:** 2021-04-27

**Authors:** Jakub Mlodawski, Marta Mlodawska, Justyna Armanska, Grzegorz Swiercz, Stanisław Gluszek

**Affiliations:** 1 Collegium Medicum, Jan Kochanowski University in Kielce, Kielce, Poland; 2 Clinic of Obstetrics and Gynecology, Provincial Combined Hospital in Kielce, Kielce, Poland

**Keywords:** Medical research, Drug development, Outcomes research

## Abstract

Induction of labour (IOL) is increasingly used in obstetric practice. For patients with unfavourable cervix, we are constantly looking for an optimal, in terms of effectiveness and safety, ripening of cervix protocol. It was retrospective cohort study. We analyzed obstetrical results in 481 patients undergoing IOL in one center using two different vaginal inserts that release prostaglandins at a constant rate for 24 h—misoprostol vaginal insert (MVI) with 200 µg of misoprostol (n = 367) and dinoprostone vaginal insert (DVI) with 10 mg of dinoprostone (n = 114). Full-term, single pregnancy patients with intact fetal membranes and the cervix evaluated in Bishop score ≤ 6 were included in the analysis. In the group of MVI patients, the labour ended with caesarean section more often (OR 2.71 95% CI 1.63–4.47) and more frequent unreassuring cardiotocographic trace indicating the surgical delivery occurred (OR 2.38 95% CI 1.10–5.17). We did not notice any differences in the percentage of vacuum extraction and patients in whom the use of oxytocin was necessary during labour induction. The clinical status of newborns after birth and the pH of cord blood did not differ between groups.The use of MVI 200 μg in patients with an unriped cervix is associated with a greater chance of completing delivery by caesarean section and increased chance of abnormal intrapartum CTG trace compared to the use of DVI 10 mg. These differences do not affect the clinical and biochemical status of the newborn.

## Introduction

Induction of labour (IOL) is a medical procedure widely used in the world. Globally, this procedure applies to every 10th pregnant woman, and in some parts of the world even every third labour is induced^1^. The indications to the IOLs are constantly being expanded. Studies on increasingly larger cohorts of patients lead to the determination of the optimal time of delivery, which is the balance of benefits and risks in cases of fetal and maternal complications. Recently published multicenter study shows that even in the case of a nulliparous woman in low risk pregnancy, induction of labour after 39 weeks of pregnancy allows for the improvement of maternal outcomes and a reduction in the caesarean section (CS) rate without worsening neonatal outcome compering to expectant management^2^. Physiologically, the cervix undergoes a ripening before delivery, which is associated with shortening, softening, changing the utero-cervical angle and opening the cervix. The Bishop score (BS) is commonly used in clinical examination to assess the cervix before delivery, which allows us to evaluate the chance of successful induction of labour^3,4^. Higher BS score before starting the IOL has strong negative correlation with the duration of the induction procedure (r = − 0.59 p < 0.01)^5^ which may increase the patient's satisfaction with the IOL^6^. Labour induction in case of favourable cervix is rather straightforward—amniotomy and oxytocin infusion. The field of discussion is only a choice between the low and high dose oxytocin protocol and the timing of the oxytocin infusion stop. In the case of patients with unfavorable cervix (low BS score), the problem is more complex due to the multitude of cervical ripening methods used. We are constantly looking for the optimal protocol of labour induction for such patients. In our study, we decided to evaluate two commercially available vaginal inserts used in pre-induction of labour: misoprostol vaginal insert (MVI) containing 200 µg of misoprostol with steady release of substance for 24 h (Misodel, Ferring Pharmaceuticals Poland) and dinoproston vaginal insert (DVI) containing 10 mg of dinoprostone released for 24 h (Cervidil, Ferring Pharmaceuticals Poland). We chose MVI 200 µg for evaluation in our study because it the only form of misoprostol registered in Poland for IOL. DVI 10 mg was selected on the basis of the same pharmaceutical form and release time. The aim of the study was to compare the above-mentioned devices in the context of obstetrical outcomes.

## Materials and methods

It was retrospective, observational, single-center cohort study. The consent for the analysis was given by the bioethical commission at the Jan Kochanowski University in Kielce (number of approval 3/21). All methods were performed in accordance with the relevant local regulations and guidelines of ethical commission.

The analysis included medical records of 481 patients who had an IOL at the Department of Obstetrics and Gynecology of the Provincial Combined Hospital in Kielce (tertiary referral hospital) between 01/02/2018 and 31/09/2020. Indications for labour induction were in line with the current recommendations of the Polish Society of Gynecologists and Obstetricians^7^. Informed consent have been obtained from all participants before start of the procedure.

The study included patients who were qualified for the IOL procedure due to medical indications and at the same time had an unfavourable cervix (BS < 7 points)^7^. All patients included were in singleton, term pregnancy (≥ 37 weeks) with fetus in cephalic presentation and no signs of labour before pre-induction applied. Exclusion criteria included all contraindications to vaginal delivery and IOL according to Polish recommendations^7^. Additionally, patients with prelabour rupture of membranes (PROM) were excluded from the study, as only one of the products (MVI) is registered in this indication. Prostaglandins in the form of slowly released vaginal inserts were used in all patients, the method of inserting the devices was in accordance with the manufacturer's instructions—we inserted product high into the posterior vaginal fornix. The patients were divided into two groups depending on the insert used. MVI group (Misodel 200 µg, Ferring Pharmaceutical Poland, n = 367) and DVI group (Cervidil 10 mg, Ferring Pharmaceutical Poland, n = 114). The disproportion in the sample size between the groups was not due to medical indications, but only to the subsequent introduction of DVI to the Polish market. The pre-induction was removed after a maximum of 24 h or when the active phase of labour began, defined as regular contractions with dilation of the cervix ≥ 4 cm. All patients from the beginning of the oxytocin infusion were subject to continuous CTG recording with computerized analysis. DVI was also removed in case of membranes rapture. In the absence of spontaneously initiated labour, oxytocin was used in the low-dose protocol for induction or augmentation of labour^8^. The amniotomy was performed with a dilatation of 4–6 cm. We considered the IOL ineffective when oxytocin infusion lasted > 12 h without achieving an active phase of labour. Arrested labor was defined as 4 h with no progression of cervical dilatation with normal uterine systolic function or no descending and no head rotation for 2 h in the second stage of labour.

We compared the groups in terms of the percentage of vaginal birth (VB), percentage of CS and vacuum extraction (VE) and the most common indications for these procedures. We also compared the groups in terms of the presence of meconium-stained amniotic fluid (MSAF), postpartum haemorrhage (PPH) and the postnatal condition of the newborn—Apgar score and umbilical cord blood pH. Statistical analysis was performed using the Statistica 13.1 program (TIBCO Software Inc.). For continuous variables we presented the arithmetic mean when the distribution was close to normal and as a median for skewed distributions. As measures of dispersion, we used standard deviations (SD) and interquartile range (IQR), respectively. Continuous variables with a distribution close to the normal meeting the assumptions about the equality of variance were compared using the Student's t-test, in the case of failure to meet the above-mentioned criteria, we used the Mann–Whitney U test. In the case of qualitative variables, we presented the data as the proportion of events in a given group and the odds ratio (OR) when applicable (MVI group vs DVI group) with 95% confidence interval, we compared the groups using Pearson’s χ2 test. In case of small expected numbers we used Yates correction. The differences were considered statistically significant in case of p < 0.05.

## Results

481 patients (MVI group = 367, DVI group = 114) were included in the analysis. The demographic characteristics of the groups are presented in Table 1. Post-term pregnancy was the most common indication for IOL, which accounted for 56% of the total indications. The groups did not differ in terms of IOL indications (Table 2). We excluded from analysis patients with the accidental insert prolapse from the vagina (5 patients in MVI group and 2 patients in DVI group), and women’s in whom insert was removed on their request (3 patients in MVI group).

**Table 1:** Demographic characteristic of study groups.

	MVI	DVI	p
Age (mean, SD)	30.17 (4.98)	29.31 (5.25)	p = 0.51
Gestational age at delivery in weeks (median, IQR)	40 (1)	40 (0.7)	p = 0.8
Multipara	36.51%	41.23%	p = 0.36
Epidural analgesia	10.90%	8.77%	p = 0.52

*SD* standard deviation, *IQR* interquartile range.

**Table 2:** Distribution of indication for IOL in particular groups.

Indication for induction of labour	MVI (n = 367)	DVI (n = 114)	Total (n = 481)	p
Post-term pregnancy [n,%]	203 (55.3%)	66 (57.9%)	269 (56%)	0.62
Gestational diabetes [n,%]	55 (14.9%)	12 (10.5%)	67 (13.9%)	0.22
Hypertensive disorders in pregnancy [n,%]	47 (12.8%)	16 (14.03%)	63 (13%)	0.73
Fetal hypotrophy (FGR/SGA) [n,%]	26 (7%)	6 (5%)	32 (6.4%)	0.49
Other indications [n,%]	36 (9.8%)	14 (12.2%)	50 (10.3%)	0.45

*FGR* fetal growth restriction, *SGA* small for gestation age.

In the studied groups, only one newborn was born in a severe condition (with Apgar < 4 points in the 1st minute of life). No newborn was in serious condition in the 5th minute of life. Therefore, we compared the groups in terms of the percentage of newborns born with Apgar ≤ 7 at 1 and 5 min of life. We analyzed difference in the median cord blood pH between groups as well as percentage of newborns born with pH < 7.1 and < 7.2. No newborns were born with a pH < 7.0 in any of the groups. Due to the low prevalence of neonatal complications and limitations resulting from the power of the study, in the case of comparing neonatal results, there is a high chance of a type II error.

The results show a greater chance of completing delivery by cesarean section in the MVI group (OR 2.71, 95% CI 1.63–4.47). In the MVI group, unreassuring fetal cardiotocographic (CTG) trace, causing CS or VE completion of labour (OR 2.38 95% CI 1.10–5.17), as well as failed induction or arrested labour being an indication for CS (OR 2.01 95% CI 1.02–3.96) and MSAF (OR 2.24 95% CI 1.01–5.11) were more often observed, however the last two results were at the borderline of statistical significance (p = 0.04). The groups did not differ in the percentage of patients who required the use of oxytocin, but the difference was at the borderline of statistical significance (p = 0.05). Due to the large amount of missing data, we do not report the percentage of uterine tachysystole in individual groups. The results of the comparative analysis are presented in Table 3.

**Table 3:** Comparison of obstetrical and neonatological outcome.

	MVI group (n = 367)	DVI group (n = 114)	p	OR (95% CI)
Cesarean section [n,%]	149 (40.60%)	23 (20.18%)	p < 0.001	2.71 (1.63—4.47)
Vacuum extraction [n,%]	9 (2.45%)	5 (4.39%)	p = 0.28	0.54 (0.18–1.67)
Oxytocin augmentation [n,%]	98 (26.70%)	41 (35.96%)	p = 0.05	0.64 (0.41–1.01)
Meconium stained amniotic fluid [n,%]	47 (12.81%)	7 (6.14%)	p = 0.04	2.24 (1.01–5.11)
Placental abruption [n,%]	5 (1.36%)	0 (0.00%)	p = 0.21	N/A
Postpartum hemorrhage [n,%]	5 (1.36%)	3 (2.63%)	p = 0.35	0.51 (0.12–2.17)
Failed induction or arrested labour (as CS indication) [n,%]	65 (17.71%)	11 (9.65%)	p = 0.04	2.01 (1.02–3.96)
Unreassuring CTG trace (as operative delivery indication) [n,%]	56 (15.26%)	8 (7.02%)	p = 0.02	2.38 (1.10–5.17)
1st minute Apgar < 8 [n,%]	29 (7.92%)	10 (18.77%)	p = 0.77	0.89 (0.42–1.89)
5th minute Apgar < 8 [n,%]	12 (3.28%)	3 (2.63%)	p = 0.73	1.25 (0.35–4.52)
pH < 7.2 [n,%]	14 (3.81%)	5 (4.42%)	p = 0.77	0.85 (0.30–2.43)
pH < 7.1 [n%]	0 (0%)	1 (0.88%)	p = 0.53	N/A
pH (median, IQR)	7.362 (0.085)	7.358 (0.093)	p = 0.65	N/A

## Discussion

The results of our study indicate an increased odds of CS in the group of patients induced with MVI 200 mcg compared to DVI 10 mg and the main reason for this difference is the increased frequency of fetal unreassuring CTG trace.

In the pivotal registration study assessing the safety and efficacy of the MVI 200 mcg (EXPADITE—multicenter, double-blind, randomised trial) in patients with unfavourable cervix. The use of MVI was associated with a shorter time from the onset of induction to vaginal delivery compared to DVI (21.5 vs 32.8 h, p < 0.001). This difference was evident in both the nulliparous and multiparous patients groups. In the MVI group, more patients had vaginal delivery within 12 h and within 24 h (19.8% vs 8.4% and 54.6% vs 34%, respectively, p < 0.001). The use of MVI was therefore characterized by a faster onset and potency, and a lower percentage of patients who needed oxytocin augmentation (48.1% vs 74.1%, p < 0.001). The caesarean section rate and the condition of the newborn did not differ between the groups, but in the group of patients who received MVI, there was a higher risk of tachysystole (RR 3.34 95% CI = 2.2–5.07), tachysystole with abnormal fetal CTG trace (RR 3.90 95% CI 2.35–6.48), and intrapartum tocolysis use (2.97 95% CI 1.96–4.50). The above-mentioned intrapartum events did not affect the neonatal outcome. Post-hoc analysis of data from the above study^10^ assessing adverse effects leading to insert removal showed that leading causes were tachysystole with FHR involvement and category II/III fetal heart rate (FHR) patterns. The inserts had to be removed due to adverse events in 11.4% of the MVI group and 4% of patients in the DVI group, respectively (p < 0.001). Removal of the insert due to adverse events did not prolong time to delivery in both study arms^10^.

The faster and stronger effect of the MVI 200 is associated with a greater risk of adverse effects. In the EXPADITE study presented above, the adverse effects did not translate into a higher risk of CS, however, in clinical practice, the rates of caesarean section and the attitudes of physicians to this procedure varies significantly between countries, therefore, the authors of this paper believe, it is difficult to extrapolate data on the risk of CS from country to country.

This problem creates space for observational studies that, despite the limitations of sample selection and randomized allocation of patients to individual groups, provide unique information from clinical practice adjusted for variance resulting from obstetric practices in a given country. All centers participating in the EXPADITE study^9^ were in the United States, and during the period, the overall caesarian sections rate in the USA was 32.8%^11^. At the time of this analysis, the average caesarean section in Poland was 43.83%^12^. According to the authors, in countries with a higher CS rate, this procedure is more frequently performed in the case of complications such as tachysystole with fetal heart involvement or category II (“suspicious” according to FIGO)^13^ fetal CTG trace. Obstetrician’s caesarian section rate is an independent variable found in predictive models that estimate IOL completion by CS^3^, and this obstetrician’s attitude toward CS we believe is country-specific and shaped by environment doctor works in.

In countries with a high basic percentage of CS, it is particularly important to limit adverse events that may result in the surgical completion of labour.

Studies show that the efficacy and risk profile of the 10 mg DVI corresponds to that of the MVI with a dose of 100 μg of misoprostol. In a study directly comparing these two inserts^14^ (DVI 10 mg with MVI 100 μg), there were no differences in the median time to vaginal delivery between the groups of patients, both in the nulliparous and parous woman groups, the percentage of CSs, intrapartum nonreassuring fetal heart rate pattern and uterine tachysystole. Neonatal outcomes were similar in both study arms^14^.

In an observational study from a Scottish center published in 2019 comparing the use of MVI 200 μg with DVI 10 mg^15^ a higher risk of tachysystole (increase in odds of 22%) and hyperstimulation (defined by tachysystole with adverse changes in the fetal heart rate pattern on CTG) was shown in the MVI group (15.6% increase of odds) compared to DVI. MVI group was characterized by reduction of median to delivery time by 77%. In both groups there was a similar percentage of caesarean sections (in our cohort the difference was significant), as mentioned earlier, this difference may be due to the high overall percentage of caesarean sections in Poland—43.83% (vs. 27.4% in the United Kingdom)^12,16^ and a more liberal approach to surgical deliveries via the abdominal route in Poland. This approach may lead to a faster CS decision in the event of CTG abnormalities more common in MVI 200 group. The authors of Scottish study^15^ showed no differences in the percentage of patients who received intrapartum oxytocin (in our analysis, the difference was on the verge of statistical significance). However, the lack of difference between the groups in this respect may be due to the labour induction protocol used, as 8% and 33% of patients in the MVI and DVI groups respectively received additional prostaglandins after removal of the inserts (p < 0.0001). In our study, oxytocin infusion was used even if the Bishop score was below 7 after 24 h. In the cited study^15^ obstetric results and pH of cord blood did not differ (however, less than 50% of newborns were analyzed for acid–base balance). The authors of the study emphasize that the lack of experience of the obstetric team with MVI may result in earlier than necessary removal of the insert from the vagina, and that tachysystole may be one of the reasons for the removal of the insert. Uterine tachystole in the EXPADITE study concerned almost 50% of patients who received MVI 200, and 20% of them have an abnormal CTG recording^9^. Overall, clinically significant tachysystole (with CTG abnormalities or requiring tocolytic therapy) were reported in 13.3% of MVI-induced patients and 4% of DVI-induced patients^9^.

The pharmaceutical form of the inserts has the advantage of being easy to remove in the event of such complications over nonrevivable forms. Resolution of tachysystole is faster with dinoprostone than misoprostol due to the difference in half-life (3 min vs. 40 min)^10^, clinically, this translates into a large difference in the median of resolution time of this complication (8.5 min for DVI and 1 h 35 min for MVI)^10^. Therefore, DVI appears to be safer in terms of both the incidence of this complication and its treatment options. It is worth noting, however, that in none of the studies found the obstetric results in the group of MVI-induced patients were worse than in the DVI^9,14,15,17^, which also applies to patients with hypertensive disorders of pregnancy^18^ and patients who experienced adverse events after using MVI or DVI^10^, although in this last case the cited analysis seems to have too little statistical power to detect differences. Relatively high safety of DVI results in attempts to use it in an outpatient setting^19^, combining it with mechanical pre-induction using a Foley catheter^20^ and in patients after CS^21^. An undoubted advantage of MVI is the registration for use during pregnancy from 36 weeks and in the case of the rupture of the membranes. DVI is not used in the latter indication due to the lack of data on the release profile of the substance in the amniotic fluid environment.

The limitation of our study was the lack of an induction to delivery time assessment. This parameter is of great importance from the pharmacoeconomic point of view. Knowing the distribution of the number of deliveries in individual hours from the start of induction can optimize the time of vaginal insertion so that the majority of deliveries take place during the daytime, with as little night-shifts as possible. This allows to reduce costs and potentially increases patient safety^22^. Research indicates that despite the fact that MVI 200 μg has a shorter interval to delivery time, which is associated with lower costs related to employing medical staff, this assumption should be adjusted for the frequency of CS performance, which brings additional costs.

Another limitation of our analysis is it observational character and the lack of adjustment of results for potential confounding factors, such as BMI, parity, and the exact BS score, but the idea behind the analysis was to reflect the general population by including all patients meeting the inclusion criteria in a given time period.

The advantage of the study is that it covers the analysis of umbilical cord blood pH in the majority of newborns (99.7%). From the epidemiological point of view, the predictive value is better than the obtained Apgar score in relation to the neurological development of children in the future^23,24^.

## Findings

The use of MVI 200 μg in patients with unfavorable cervix is associated with an increased likelihood of CS compared to the use of DVI 10 mg, and the main reason for this difference is the increased risk of abnormal CTG recording in this group of patients.

Clinical status and pH of umbilical cord blood does not differ between groups.

## Data Availability

The data that support the findings of this study are openly available in OSF Storage at https://doi.org/10.17605/OSF.IO/ECYU9.
